# Suberin in plants: biosynthesis, regulation, and its role in salt stress resistance

**DOI:** 10.3389/fpls.2025.1624136

**Published:** 2025-06-30

**Authors:** Ruonan Chen, Pengrui Wang, Jianing Liu, Xue Yang, Xiaoying Gong, Hongliang Zhou, Ning Han, Zhen Yang

**Affiliations:** Shandong Provincial Key Laboratory of Microbial Engineering, School of Biologic Engineering, Qilu University of Technology (Shandong Academy of Sciences), Jinan, Shandong, China

**Keywords:** suberin, salt stress, salt resistance, biosynthesis, transportation, salt exclusion

## Abstract

Soil salinization represents a significant global ecological challenge. Plants encounter salt stress in their growth environments. Suberin, a hydrophobic polymer, plays a critical role in plant salt tolerance. This review examines the mechanisms by which suberin contributes to salt tolerance. Suberin comprises polyaliphatic and polyphenolic domains. Its biosynthesis involves multiple enzymes, including fatty acid synthases, the fatty acid elongation complex, and various cytochrome P450 monooxygenases. ABCG transporters and lipid transfer proteins facilitate the transport of suberin monomers from the endoplasmic reticulum to the plasma membrane and cell wall. Plants utilize suberin lamellae to respond to salt stress through multiple mechanisms. Under salt stress, the structure and composition of suberin lamellae undergo modifications, including increased thickness and enhanced very-long-chain fatty acid components. In addition, salt stress elevates the expression of genes associated with suberin biosynthesis and transport. Mutations in these genes often result in salt-sensitive phenotypes. Fundamentally, suberin contributes to forming the hydrophobic component of the apoplastic barrier, thereby reducing passive Na^+^ influx and restricting sodium uptake to protect plants from ion toxicity. Understanding the mechanisms of suberin in salt tolerance offers potential strategies for enhancing crop salt tolerance through genetic engineering.

## 1 Introduction

Plants have developed regulatory processes to respond and adapt to the dynamic conditions of complex and variable terrestrial environments (Shams and Khadivi, 2023). Roots are particularly affected by soil abiotic stresses such as salt and drought. The regulation of root water absorption and ion selectivity constitutes the initial defense mechanism against plant stress. Root suberin lamellae have demonstrated significant importance in plant stress resistance by functioning as an apoplastic barrier that modulates the diffusion of aqueous solutes, gases, and water (Serra and Geldner, 2022).

## 2 The formation of suberin in plants

Suberin is a complex hydrophobic polymer consisting of polyaliphatic and polyphenolic domains. Two types of suberin lamellae have been identified based on electron density in electron microscopy, electron-dense lamellae (appearing dark) and electron-lucent lamellae (appearing semitransparent). This distinction stems from the chemical attributes, with the polyaliphatic domain primarily associated with the electron-lucent lamellae and the polyphenolic domain to be associated with the electron-dense lamellae (Graca and Santos, 2007). The polyaliphatic domain consists of very-long-chain fatty acids (VLCFAs), including ω-hydroxyacids, dicarboxylic acids and primary alcohols. These aliphatic components integrate into a polymeric matrix anchored to a glycerol backbone, establishing a hydrophobic and structurally diverse platform within the suberin macromolecule. The polyphenolic domain contains *p*-hydroxycinnamic acid derivatives, predominantly ferulic acid. These phenolic components contribute to the cross-linking and recalcitrance of the suberin polymer, providing enhanced resistance to chemical and biological degradation (Nomberg et al., 2022).

### 2.1 The biosynthesis of suberin monomers

**Figure 1:**
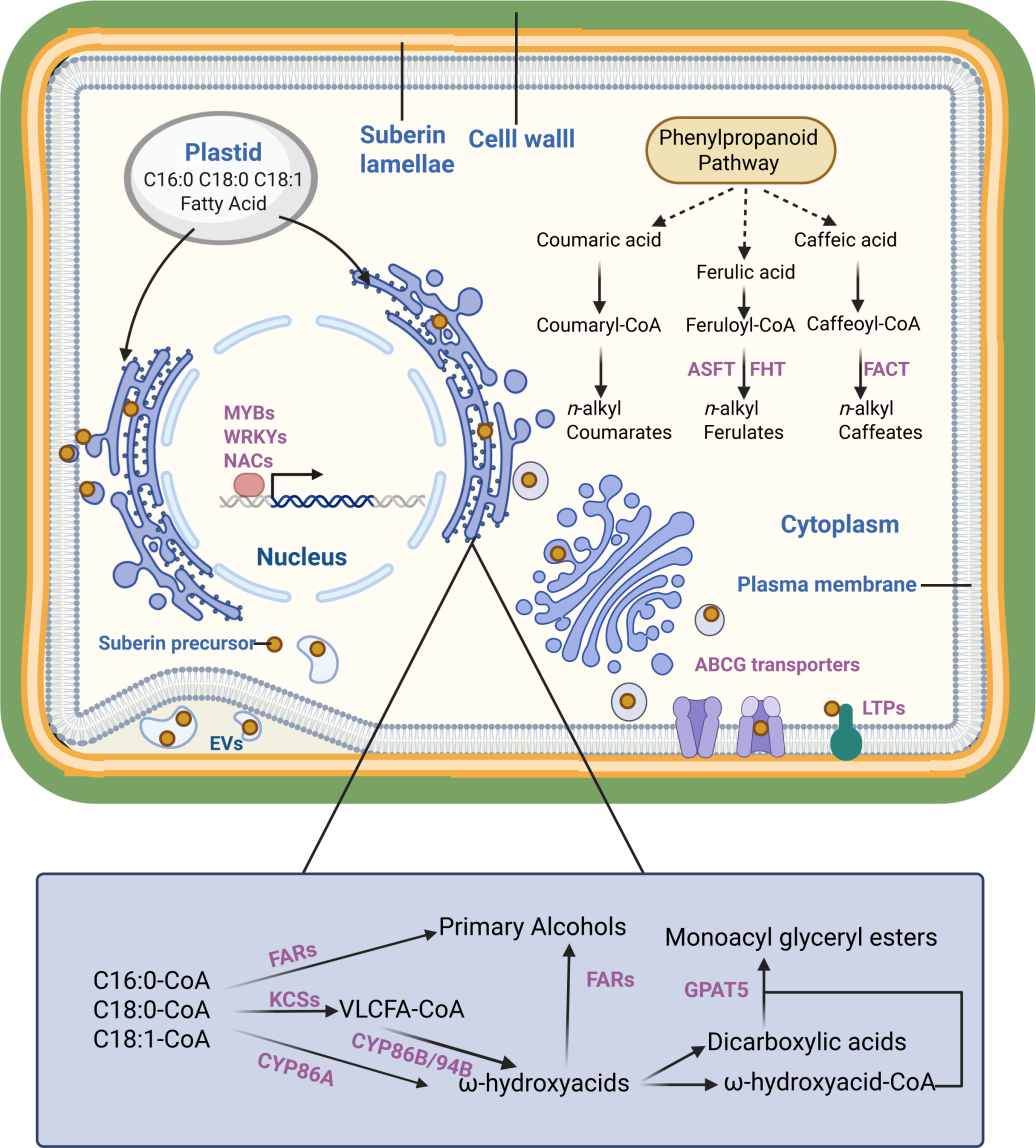
The formation of suberin lamellae in plants. The scheme is proposed based on previous studies. It presents suberin monomer (yellow dot) in plant cell, enzymes/transporters involved in suberin monomer biosynthesis, transportation and regulation (purple). Dashed lines represent more than one enzymatic step.

Suberin biosynthesis is initiated with fatty acid synthesis. In plants, C16:0, C18:0 and C18:1 fatty acids are synthesized in plastids by the fatty acid synthase complex. These compounds enter the endoplasmic reticulum (ER) after the CoA group is added. C16:0-CoA, C18:0-CoA and C18:1-CoA form VLCFAs by fatty acid elongation complex (FAE) in the ER. The β-ketoacyl-CoA synthase (KCS) enzymes perform essential functions in this process, catalyzing the rate-limiting step and determining product length. KCS2/DAISY and KCS20 are essential enzymes for suberin polyaliphatic monomer biosynthesis in *Arabidopsis thaliana* (Lee et al., 2009; Franke et al., 2009). The *atkcs2/daisy* mutant displayed notable changes in suberin composition with decreased C22 and enriched C16, C18 and C20 derivatives. In addition, the *atkcs20kcs2* double mutant exhibited abnormal endodermal suberin lamellae. Chemical analysis revealed significantly reduced C22 and C24 VLCFA levels, while C20 VLCFA derivatives accumulated excessively (Lee et al., 2009). Similar results have been reported in in potato (*Solanum tuberosum*). *StKCS6* silencing in the tuber periderm reduced the levels of VLCFAs exceeding C28 in length (Serra et al., 2009a). All these indicated that the KCS family is closely related to the elongation of fatty acid which act as monomers of suberin lamella (**Figure 1**).

Compared to unmodified fatty acids, ω-hydroxy acids and α, ω-diacids are crucial for suberin polyester extension (Serra and Geldner, 2022). Cytochrome P450 monooxygenases are essential enzymes in ω-hydroxylate fatty acid biosynthesis in plants. The *atcyp86a1/horst* mutants exhibited significantly reduced levels of ω-hydroxy acids in roots compared with the wild type (Hofer et al., 2008). Similarly, CYP86B1/RALPH has been identified as a critical oxidase for suberin monomer ω-hydroxy acid and α,ω-dicarboxylic acid biosynthesis in *Arabidopsis*, with *atcyp86b1/ralph* mutants demonstrating a substantial reduction in C22 and C24 ω-hydroxy acid and α,ω-dicarboxylic acid contents (Molina et al., 2009; Compagnon et al., 2009). In potato, *CYP86A33* silencing led to a 60% decrease in aliphatic suberin, particularly affecting C18:1 ω- hydroxyacid (70%) and α,ω-dicarboxylic acids (90%) levels (Serra et al., 2009b). In addition to CYP86s, CYP94s have also been reported to be closely related to fatty acid ω-hydroxylation process. The *atcyp94b1* mutant exhibited marked reduction in ω-hydroxy acid and α,ω-dicarboxylic acid levels. Conversely, heterologous *AoCYP94B1* overexpression in *Arabidopsis* resulted in enhanced accumulation of C18 octadecanol C16 ω-hydroxy acids, and C16 α,ω-dicarboxylic acids compared with the wild type (Krishnamurthy et al., 2020). Similar outcomes were observed with *AoCYP94B3* (Krishnamurthy et al., 2021).

Primary alcohols and glycerol constitute essential components of suberin polyaliphatic monomers. The fatty acyl reductases facilitate the reduction in α-carboxylic groups of VLCFA-CoA to generate suberin monomer primary alcohols. *Arabidopsis* mutants *atfar1, atfar4*, and *atfar5* showed modified suberin composition in the root and seed coat, with decreased levels of C22, C20 and C18 primary alcohols, respectively (Domergue et al., 2010).

The glycerol-3-phosphate acyltransferase (GPAT) transfers the acyl-CoA to glycerol-3 phosphate. GPAT5 demonstrates broad substrate specificity with the CoA-derivatives of C16–C22 fatty acids, ω-hydroxy acids, and α,ω-dicarboxylic acids. It is an essential role in suberin synthesis is evidenced by the *atgpat5* mutant, which showed a 50% reduction in aliphatic suberin in roots and significantly decreased the suberin-related dicarboxylic acid and ω-hydroxy acid contents of seed coats (Yang et al., 2012; Beisson et al., 2007).

Ferulic acid, synthesized by phenylpropanoid pathway, is the primary aromatic monomer of suberin (Graça, 2015). Its significance in suberin formation was demonstrated through phenylpropanoid pathway inhibition studies, which showed blocked suberin deposition that can be restored by exogenous ferulic acid application (Andersen et al., 2021). Aliphatic suberin feruloyl transferase (ASFT) mediates the transfer of feruloyl-CoA acyl groups to ω-hydroxy acids and primary alcohols, a crucial step in suberin monomer biosynthesis. *Arabidopsis atasft* mutants demonstrated near-complete absence of ferulic acid in their polyphenolic domain affecting aliphatic suberin monomer levels (Molina et al., 2009; Gou et al., 2009). Furthermore, ENHANCED SUBERIN1 (ESB1) is involved in suberin synthesis, as evidenced by the *Arabidopsis* mutant *atesb1* showing significantly increased root suberin content (Baxter et al., 2009).

### 2.2 The transport of suberin monomers

While numerous enzymes involved in suberin monomer synthesis or modification are located in the ER lumen or at the cytosolic face of the ER, suberin lamellae formation typically occurs on the plasma membrane surface or cell wall. The transport of suberin monomers from the ER to the plasma membrane and their transmembrane transport are essential steps in suberin lamellae formation (Serra and Geldner, 2022). The mechanisms underlying these processes remain incompletely understood, and several hypotheses exist regarding them. Lipid-like suberin monomers synthesized at the ER may transfer directly through the ER-plasma membrane contact site. Vesicle trafficking by the Golgi and trans-Golgi networks represents another potential mechanism, given its established role in cuticular wax delivery (Mcfarlane et al., 2014). Additionally, earlier studies documented plasma membrane invaginations containing extracellular tubules or vesicles. These structures were initially proposed as intermediates in lipid-like substance transport, including suberin monomers (Scott and Peterson, 1979; Peterson and Ma, 2001). This concept remained dormant until recent research identified similar structures called extracellular vesicular-tubular (EVs), which demonstrate a strong correlation with suberin lamellae formation. The accumulation of large numbers of EVs in suberizing cells were observed through electron-microscopy (De Bellis et al., 2022).

After suberin monomers reach the plasma membrane, they are transported across the membrane to the apoplast. Several ABC transporters of the G-clade (ABCG) have been implicated in this process. The *Arabidopsis* DSO/ABCG11 transporter influences cutin metabolism in reproductive organs and suberin content of roots (Panikashvili et al., 2010). In addition, three Arabidopsis transporters (ABCG2, ABCG6, and ABCG20) participate in suberin metabolism in roots and seed coats (Yadav et al., 2014; Fedi et al., 2017). The triple mutant of these genes exhibited changes in suberin lamellae structure, composition, and properties in the root and seed coat (Yadav et al., 2014). Further research confirmed AtABCG1’s role in suberin transport, as the *atabcg1* mutant showed decreased root suberin content, particularly in VLCFAs, primary alcohols, and dicarboxylic acids (Shanmugarajah et al., 2019). Furthermore, homologs of these genes, such as *StABCG1* and *OsABCG5/RCN1*, facilitate suberin precursor transport (Landgraf et al., 2014; Shiono et al., 2014). Beyond ABCG transporters, the LIPID TRANSFER PROTEIN (LTP) superfamily contributes to cuticular wax deposition and pollen wall formation (Chen et al., 2022; Gao et al., 2021; Edqvist et al., 2018). Evidence indicates that LTPs participate in suberin monomers transport. *AtLTPI-4* contributes to suberin formation in *Arabidopsis* crown galls, as the *atltpi4* mutant showed significantly reduced suberin accumulation. Protein expression in epidermal cells increased C24 and C26 VLCFA levels (Deeken et al., 2016). In addition, Arabidopsis *LTPG15*, expressed in the root endodermis and seed coat, facilitates suberin monomer transport (Lee and Suh, 2018). Collectively, both ABCG transporters and LTPs may transport suberin monomers across the membrane to the apoplast where suberin lamellae form.

### 2.3 Regulation of suberin biosynthesis

Suberin deposition exhibits cell and tissue specificity and responds to various environmental stresses, reflecting strict transcriptional regulation of suberin biosynthesis-related genes. The MYB family regulates suberin biosynthesis and deposition across species and tissues (Cesarino, 2022). *AtMYB41* activates aliphatic suberin synthesis and deposition. *AtMYB41* overexpression enhanced the transcription of suberin biosynthesis genes in leaves and induced suberin deposition on leaf cell walls, forming suberin-lamellae-like structures (Kosma et al., 2014). Subsequently, additional MYB transcription factors involved in suberin biosynthesis regulation were identified and functionally validated. MdMYB93 demonstrated a significant role in regulating suberin deposition in russeted apple (*Malus domestica*) skins (Legay et al., 2016). MYB9 and MYB107 regulate suberin deposition in seed coats and fruit skins (Lashbrooke et al., 2016). Shukla et al. identified four MYB transcription factors (MYB41, MYB53, MYB92, and MYB93) that individually respond to developmental and exogenous signals and promote endodermal suberin formation (Shukla et al., 2021). In potato wound-healing tissues, StMYB102 and StMYB74 function as regulators of wound suberin biosynthesis and deposition (Wahrenburg et al., 2021).

The NAC and WRKY families serve as transcriptional regulators of suberin biosynthesis. *ANAC046* expression occurs primarily in the root endodermis and periderm, with wound-induced expression in leaves. *ANAC046* overexpression enhanced the expression of suberin biosynthesis genes in the roots and leaves, increasing root wax and suberin accumulation. This indicated that *ANAC046* functions as a key transcription factor promoting suberin biosynthesis in *Arabidopsis* roots (Mahmood et al., 2019). StNAC103 and StNAC101 act as suberin biosynthesis repressors in potato, evidenced by increased suberin and wax in RNAi-mediated mutants (Soler et al., 2020). WRKY33 functions as an upstream regulator of CYP94B1 in *Arabidopsis*, with *atwrky33* mutants showing reduced suberin and salt-sensitive phenotypes (Krishnamurthy et al., 2020). Krishnamurthy et al. identified AtWRKY9 as a suberin biosynthesis regulator through its control of *AtCYP94B31* and *AtCYP86B1* expression in *Arabidopsis* (Krishnamurthy et al., 2021).

Various hormones, including abscisic acid (ABA) (Shiono et al., 2022; Shiono and Matsuura, 2024), ethylene (Liu et al., 2021), auxin (Ursache et al., 2021), and gibberellin (Binenbaum et al., 2023) participate in regulating suberin biosynthesis.

## 3 The role of suberin in salt stress resistance

Salt stress is a major environmental challenge affecting plant growth and production globally (Zhou et al., 2024; Shams et al., 2024, 2023). Plant survival under salt stress depends on maintaining low cytoplasmic Na^+^ concentration in shoots. Apoplastic transpiration bypass flow of water and solutes contributes substantially to Na^+^ entry into shoots (Ochiai and Matoh, 2002). However, suberin lamellae in the endodermis and exodermis can block this bypass flow of water and solutes (Hose et al., 2001; Steudle and Peterson, 1998). This characteristic establishes a strong connection between suberin lamellae and plant salt tolerance.

### 3.1 The structure and composition of suberin lamellae change in response to salt stress

Suberin lamellae serve as a natural barrier that restricts Na^+^ transport from roots to shoots through the bypass flow. Its thickness and location directly influence the effectiveness of salt exclusion in plants. Three rice (*Oryza sativa*) varieties cultivated under varying salt concentrations demonstrated increased root suberization and upregulation of suberin synthesis genes in response to salt stress. The formation of both the endodermal and exodermal suberin lamellae advanced toward the root tip, indicating accelerated root suberization under salt stress. The variety exhibiting the highest degree of root suberization displayed the lowest Na^+^ accumulation in shoots (Krishnamurthy et al., 2009, 2011). Similar observations were documented in *Avicennia officinalis*, where salt treatment increased root suberin lamellae thickness and significantly reduced Na^+^ transport to aboveground parts by the xylem (Krishnamurthy et al., 2014). Subsequent research using corn (*Zea mays*), olive (*Olea europaea*), and grape (*Vitis vinifera*) also showed salt stress induced thickening of the suberin lamellae (Rossi et al., 2015; Shen et al., 2015; Wang et al., 2023). These findings demonstrate that salt stress promotes suberin deposition and thickening, effectively inhibiting Na^+^ absorption and transportation, thereby establishing an inverse relationship between Na^+^ permeability and root suberization.

In addition to the change of the structure, suberin composition undergoes changes under salt stress. NaCl treatment induced a 22% increase in total suberin content in *Arabidopsis* after 100 mM NaCl exposure, with significant increases in dicarboxylic fatty acids and modest increases in 18:0 ferulate and 20:0 and 22:0 coumarates (De Silva et al., 2021). In *Chenopodium album*, salt stress significantly increased saturated and unsaturated VLCFAs with chain lengths of C20–C26 (Ivanova et al., 2016). Moreover, increasing saturation and length of fatty acids represents an adaptation strategy against salt stress in halophytes (Sui et al., 2018). These findings indicate that salt stress enhances VLCFA components in plant suberin, potentially strengthening the hydrophobic barrier, limiting Na^+^ flux, and maintaining membrane stability.

### 3.2 Expression of genes related to suberin biosynthesis were induced by salt stress

The structural changes in suberin lamellae structure under salt stress indicate the regulation of suberin synthesis and transport-related gene expression. Transcriptome analyses reveal the salt stress-induced transcription of biosynthesis genes (Wei et al., 2020; Yang et al., 2018). In rice, an increased transcript levels of suberin biosynthesis gene was detectable as early as 30 minutes after NaCl treatment (Krishnamurthy et al., 2009). Analysis of tissue-specific differential induction revealed that the expression of suberin-related genes in roots correlates most strongly with salt stress. In quinoa (*Chenopodium quinoa*), *CqGPAT5a* and *CqGPAT5b* demonstrated high expression in roots with rapid induction under high salt stress (Wang et al., 2025b). Similarly, *VvKCS11* exhibits root-specific high expression and strong salt stress induction in grape (Yang et al., 2020). Salt treatment increases the expression of *CYP94B3* and *CYP86B1*, key suberin precursor synthesis genes, in the roots of both *Arabidopsis* and medicinal plants (Krishnamurthy et al., 2021).

Studies using suberin-related gene mutants further demonstrate suberin’s significance in plant salt tolerance. In wheat (*Triticum aestivum*), *TaGPAT6* overexpression enhanced suberin deposition in the seed coat and root tip outer layers, improving salt tolerance through reduced Na^+^ accumulation. Conversely, *tagpat6* mutants exhibited decreased suberin deposition, enabling Na^+^ accumulation and resulting in salt sensitivity (Wang et al., 2025a). The *Arabidopsis* mutant *cyp86a1* displayed salt sensitivity with increased Na^+^ and decreased K^+^ accumulation (Wang et al., 2020). Furthermore *atmyb107* and *atmyb9* mutants showed a significantly reduced seed suberin monomer content, leading to decreased germination rates under salt stress (Lashbrooke et al., 2016). Similarly, *ABCG23* mutation reduced C24 ω-hydroxy fatty acids and 1, ω-dicarboxylic acids in the mutant seed coats, diminishing germination rates under salt stress (Kim et al., 2025). These mutant studies demonstrate that altered suberin composition or lamellae structure affects the ion and water absorption, often resulting in salt-sensitive phenotypes (De Silva et al., 2021).

## 4 Conclusions

**Figure 2:**
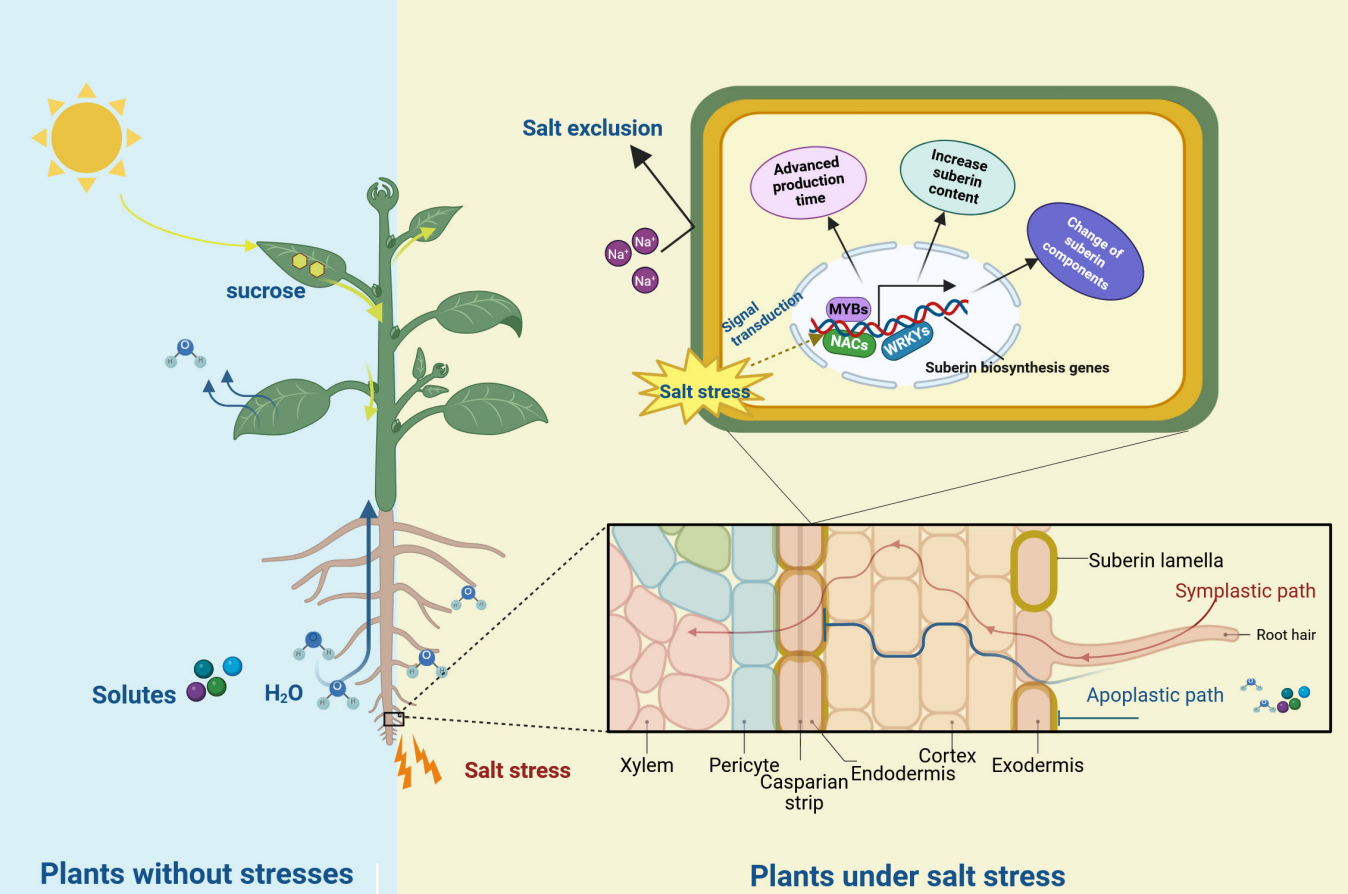
The role of suberin in plant in response to salt stress. The light blue background represents the non-stress environment, and the yellow background represents the salt-stress environment. Under salt stress, genes related to suberin lamellae foemation are induced by the regulation of transcription factors (such as MYBs, NACs and WRKYs), resulting in the advanced production time, the change of components and the increased content of suberin. The suberin lamellae (yellow circles) in roots was induced and block the bypass flow of Na^+^.

Suberin functions as a natural hydrophobic barrier in plants. Salt stress induces the expression of genes related to suberin biosynthesis and transportation were induced by salt stress and transport, modifying suberin composition and lamellae structure. Increased suberin content, altered suberin monomer composition, and thickened lamellae enhance the hydrophobic barrier. This barrier can thus restrict sodium uptake, protecting plant photosynthetic organs from ion toxicity (**Figure 2**). The role of suberin in salt stress response suggests potential applications in genetic engineering to enhance suberin deposition for developing salt-tolerant crops.
